# Lower extremity infections: Essential anatomy and multimodality imaging findings

**DOI:** 10.1007/s00256-024-04567-w

**Published:** 2024-01-20

**Authors:** George R. Matcuk Jr., Matthew R. Skalski, Dakshesh B. Patel, Brandon K. K. Fields, Leah E. Waldman, Paolo Spinnato, Ali Gholamrezanezhad, Sanaz Katal

**Affiliations:** 1 Department of Imaging, Cedars-Sinai Medical Center, 8700 Beverly Blvd, Ste M-335, Los Angeles, CA 90048 USA; 2 Department of Radiology, Palmer College of Chiropractic – West Campus, San Jose, CA 95134 USA; 3 Department of Radiology, Keck School of Medicine, University of Southern California, Los Angeles, CA 90033 USA; 4 Department of Radiology & Biomedical Imaging, University of California, San Francisco, San Francisco, CA 94143 USA; 5 Department of Radiology, Duke University School of Medicine, Durham, NC 27705 USA; 6 Diagnostic and Interventional Radiology, IRCCS Istituto Ortopedico Rizzoli, 40136 Bologna, Italy; 7 Melbourne, Australia

**Keywords:** Lower extremity infection, Cellulitis, Septic arthritis, Abscess, Osteomyelitis, Imaging

## Abstract

In modern practice, imaging plays an integral role in the diagnosis, evaluation of extent, and treatment planning for lower extremity infections. This review will illustrate the relevant compartment anatomy of the lower extremities and highlight the role of plain radiographs, CT, US, MRI, and nuclear medicine in the diagnostic workup. The imaging features of cellulitis, abscess and phlegmon, necrotizing soft tissue infection, pyomyositis, infectious tenosynovitis, septic arthritis, and osteomyelitis are reviewed. Differentiating features from noninfectious causes of swelling and edema are discussed.

## Introduction

Skin and soft tissue infections are a common cause for patients to seek medical care, with a 65% increase in total healthcare visits in the USA from 8.6 million in 1997 to 14.2 million in 2005. This translates to an incidence of 48.5 infections per 1000 patient-years in 2010, and accounted for 2.0% of all hospital admission in 2011 [1]. These include conditions such as cellulitis (56.8%), abscesses (35.1%), ulcers (10.3%), surgical wounds (8.9%), diabetic wounds (6.6%), nondiabetic wounds (3.6%), necrotizing fasciitis (2.0%), and others (4.4%). Likewise, there was an increase in the annual incidence of osteomyelitis from 11.4 cases per 100,000 person-years from 1969 to 1979 to 24.4 per 100,000 person-years from 2000 to 2009, with this incidence tripled in patients older than 60 years [2].

Imaging plays an important role in the diagnosis of lower extremity infections, evaluation of the extent of disease, and treatment planning. This article will review the relevant compartment anatomy of the lower extremities and the role of plain radiographs, CT, US, MRI, and nuclear medicine in the diagnostic workup. We will discuss the imaging features of cellulitis, abscess and phlegmon, necrotizing soft tissue infection, pyomyositis, infectious tenosynovitis, septic arthritis, and osteomyelitis.

## Relevant anatomy

Localizing soft tissue infections to the anatomic compartment(s) of involvement can help predict the extent of spread and guide surgical intervention and management [3]. In rare cases, infection may be complicated by compartment syndrome as swelling escalates [4]. There is a superficial (membranous) fascia within the subcutaneous tissues, with fat both superficial and deep to this layer [5]. The deep fascia is comprised of the investing layer covering the superficial aspect of the muscles (not to be confused with the superficial layer of fascia) and the deep intermuscular fascia. Understanding compartment anatomy also plays a role in diagnosing necrotizing fasciitis, which typically involves 3 or more compartments in one extremity, with extensive involvement of the deep fascia [6].

An understanding of anatomy may also help to explain potential routes of spread of infection between structures that may be contiguous. For example, foot infections can spread into the deep compartment of the leg along the tendon sheaths of the long flexors, as well as into the tibiotalar and posterior subtalar joints (and vice versa), as these commonly communicate [7].

### Compartments of the thigh

**Fig. 1:**
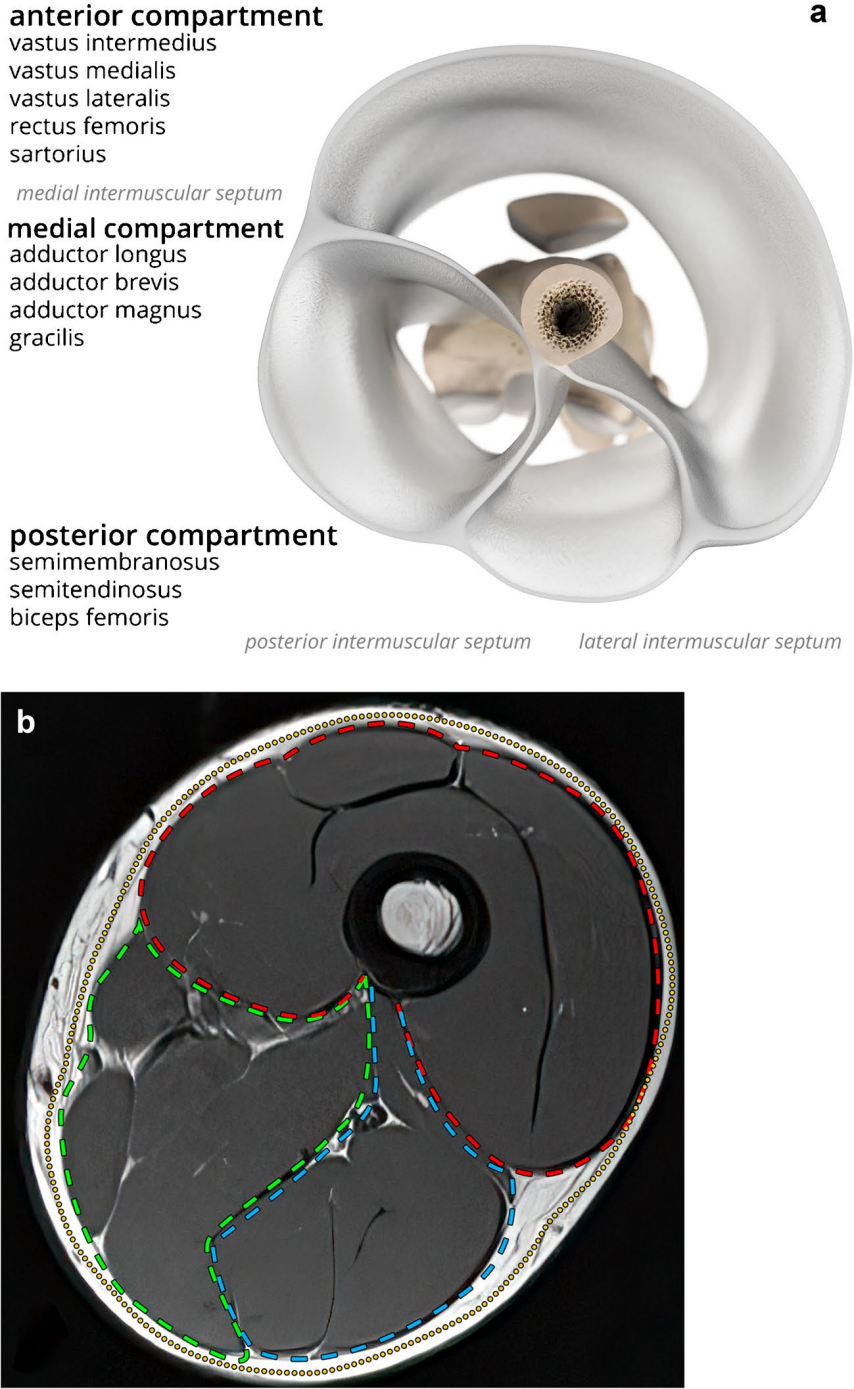
Axial illustration (**a**) and axial T1 MRI (**b**) and of the thigh compartment anatomy. The labels in **a** indicate the muscles contained in each compartment. The dashed lines in **b** indicate the superficial (yellow) and anterior (red), medial (green), and posterior (blue) compartment fascia

The thigh is composed of three compartments: anterior, medial, and posterior (Fig. 1) [8]. The medial intermuscular septum separates the anterior from the medial compartment. The posterior intermuscular septum separates the medial from the posterior compartment. The lateral intermuscular septum separates the posterior from the anterior compartment. The anterior compartment contains the knee extensor musculature, along with the femoral artery, femoral vein, and saphenous nerve. The anterior compartment wraps around the lateral thigh to border the posterior compartment. The posterior compartment contains the knee flexor muscles (hamstrings) and sciatic nerve. The medial compartment contains the adductor musculature and deep femoral artery and vein.

### Compartments of the leg

**Fig. 2:**
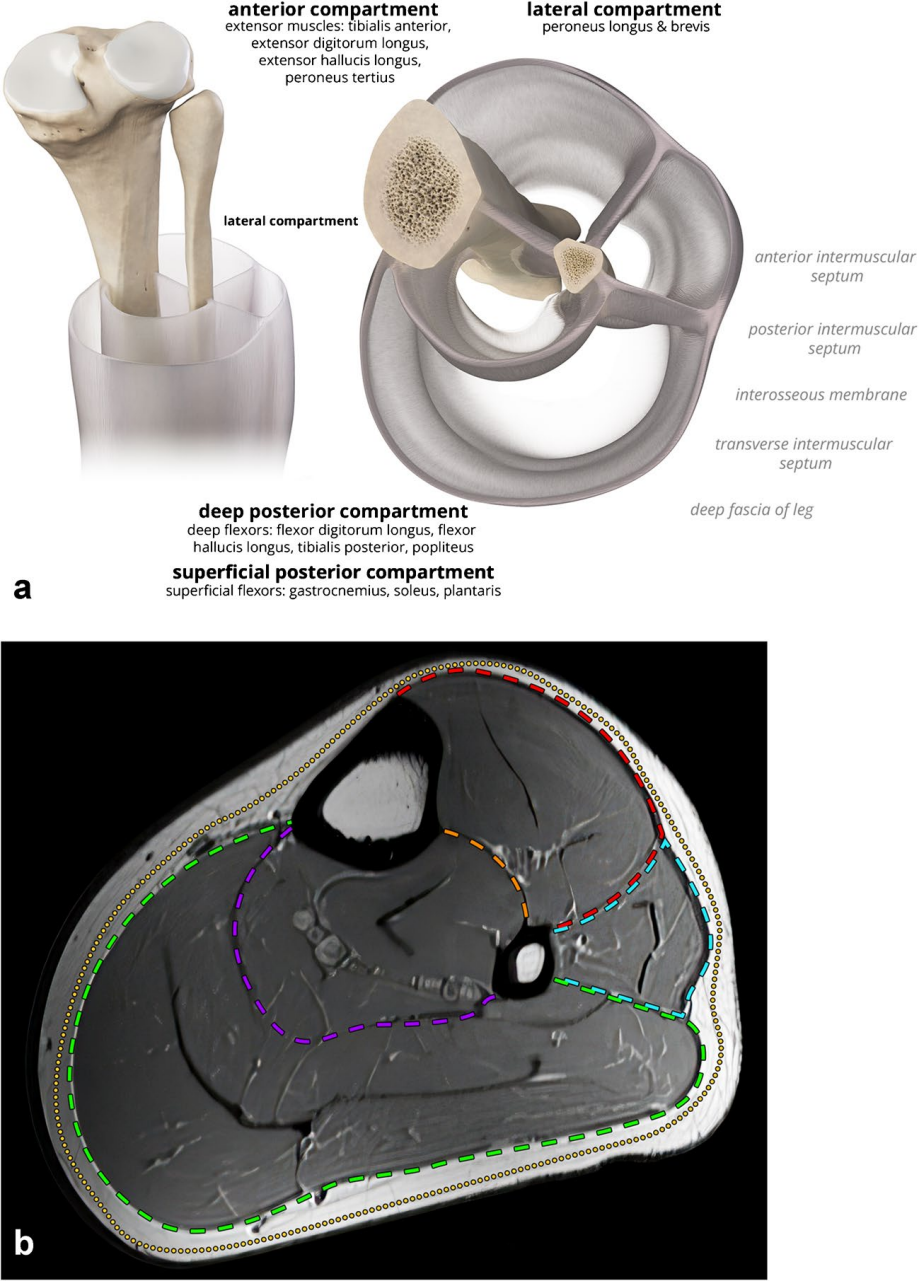
Posterior and axial illustration (**a**) and axial T1 MRI (**b**) of the leg compartmental anatomy. The black labels in **a** indicate the muscles contained in each compartment. The gray labels in **a** indicate the name of the fascial structure. The dashed lines in **b** indicate the superficial (yellow) and anterior (red), lateral (blue), and deep posterior (purple) and superficial posterior (green) compartment fascia and intermuscular septum (orange)

The leg contains four compartments and is contained by the deep/crural fascia, which encircles the leg and affixes it to the anteromedial tibia (Fig. 2) [8, 9]. From the deep fascia, the anterior and posterior septa arise laterally, dividing the lateral compartment from the anterior and superficial posterior compartments. The deep (posterior) compartment is contained by the interosseous membrane anteriorly and transverse intermuscular septum posteriorly, which separates it from the anterior and superficial posterior compartments, respectively. The anterior compartment is the most common to suffer compartment syndrome. It contains the extensor musculature along with the deep peroneal nerve and anterior tibial vessels. The lateral compartment contains the peroneal musculature and superficial peroneal nerve. The superficial posterior compartment contains the ankle flexors and median cutaneous nerve. The deep compartment contains the flexor digitorum longus (FDL), flexor hallucis longus (FHL), tibialis posterior, and popliteus muscles, along with the tibial nerve, posterior tibial vessels, and peroneal vessels.

### Compartments of the foot

**Fig. 3:**
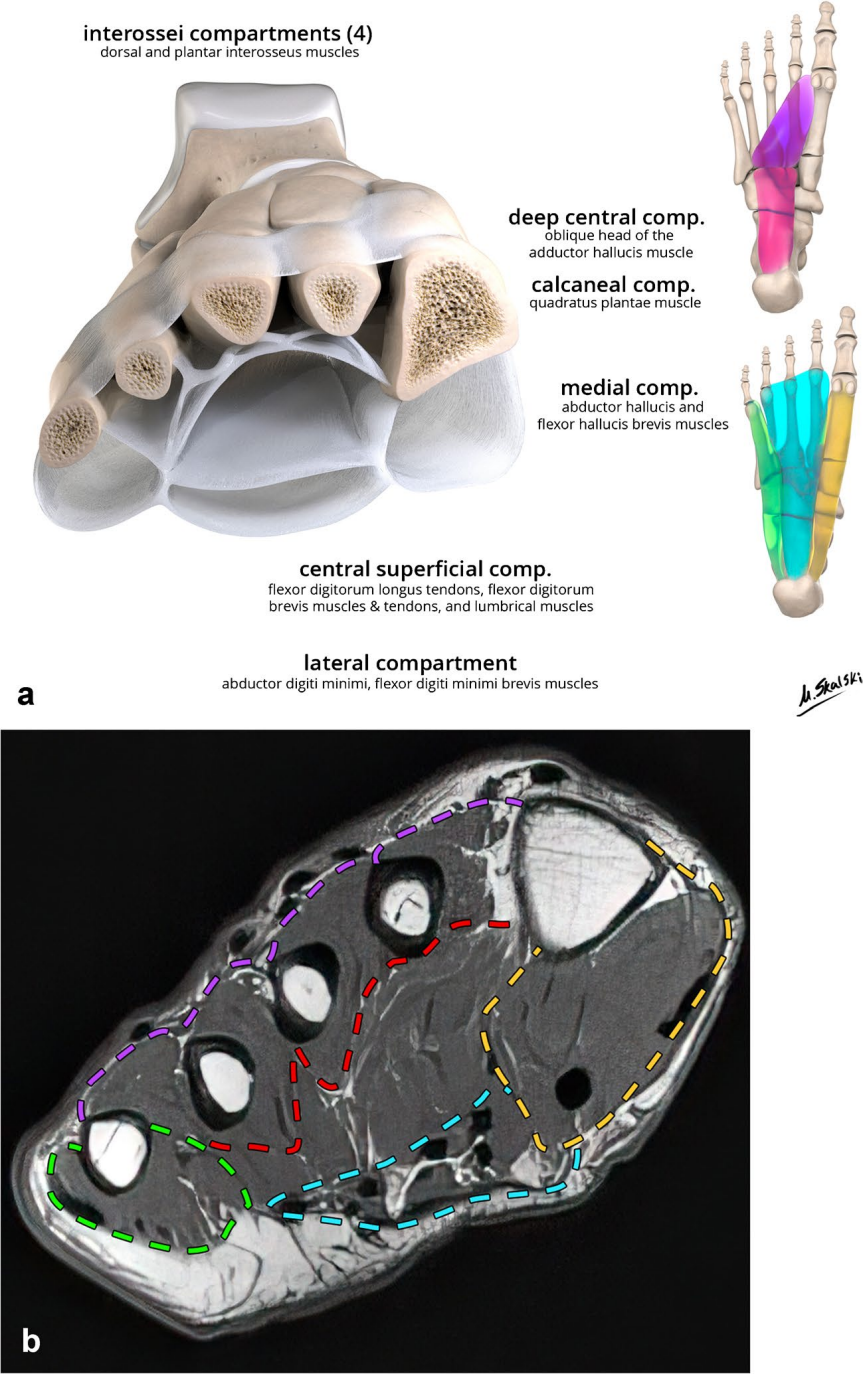
Coronal and plantar illustrations (**a**) and short axis T1 MRI (**b**) of the foot compartment anatomy. The compartments are labeled along with their contained muscular anatomy in **a**. The dashed lines in **b** indicate the superficial medial (yellow), central (blue), and lateral (green) with the deep central compartment between the red and blue lines, interosseous compartments between the red and purple lines, and dorsal compartment superficial to the purple line

The foot contains several compartments, as illustrated in Fig. 3 [9, 10]. Most surgically accessible are the four interossei compartments, which contain the dorsal and plantar interosseous muscles. There are two deep compartments consisting of the calcaneal compartment proximally, which contains the quadratus plantae muscle, and the deep central compartment more distally, which contains the adductor hallucis muscle. Overlying the deep compartments is the central superficial compartment, which contains the flexor digitorum longus tendons, flexor digitorum brevis muscle and tendons, and the lumbricals. On either side of this are the lateral and medial compartments. The lateral compartment contains the abductor and flexor digiti minimi brevis muscles, while the medial compartment contains the abductor hallucis and flexor hallucis brevis muscles. Some authors include a dorsal compartment containing the extensor digitorum brevis and extensor hallucis brevis muscles [11].

## Imaging modalities

Imaging plays a critical role in the diagnosis and management of lower extremity infections as it confirms clinical suspicion, identifies predisposing or associated conditions, localizes infection sites, and determines the extent of involvement. Conventional radiographs (CRs) are usually the initial modality obtained and are useful in detecting structural deformities (as can be seen with septic arthritis and acute and chronic osteomyelitis), surgical changes (including hardware-related infection), and other possible differential considerations such as fractures that may present with similar clinical features [12]. CR is also useful for demonstrating radiopaque foreign bodies and vascular calcifications as a marker of peripheral arterial disease. However, while they provide an overview of the region of interest, they have limited sensitivity in detecting early infections. Hence, regardless of CR results, additional imaging is typically required for further evaluation, except in cases where a clinical diagnosis (e.g., uncomplicated cellulitis) is sufficient.

Computed tomography (CT) can provide comparable and additional information to radiographs with the added benefit of reconstruction capabilities [13]. CT is a rapid imaging tool frequently used in the emergency department to assess potential musculoskeletal infections, as it is invaluable for detecting deep complications of cellulitis, soft tissue abscesses, gas, and small foreign bodies [12, 14]. While CT may not be as effective as MRI or radionuclide imaging in detecting intramedullary osteomyelitis at an early stage, it can detect periostitis and bone rarefaction during the later stages of infection. In osteomyelitis, CT findings typically include soft tissue swelling, periosteal reaction, decreased attenuation of the medullary space, and cortical erosions. Similarly, in septic arthritis, CT usually demonstrates joint effusion, articular narrowing, and marginal erosion. Notwithstanding these advantages, determining the extent of involvement within the bone and soft tissues using CT remains limited. However, CT is rapid and easily accessible and therefore often used in the initial evaluation of infection or where MRI is not available, feasible, or where susceptibility artifact from metallic implants severely limits evaluation.

Ultrasound (US) is usually not the primary imaging modality obtained for the evaluation of suspected lower extremity infections, although its indications for use are increasing, particularly in the emergency department. It can effectively visualize superficial fluid collections, joint effusions, and subperiosteal abscesses, which may be advantageous in specific cases, such as in pediatric patients [15].

Radionuclide imaging is also helpful in evaluating lower extremity infections, particularly in cases with extensive hardware or to differentiate osteomyelitis from neuropathic arthropathy. A three-phase bone scan is sensitive but not specific. A radiolabeled leukocyte scan is both sensitive and specific, making it the preferred radionuclide test for osteomyelitis, with an accuracy of approximately 90% when combined with sulfur colloid imaging [12]. Recent studies have shown that a radiolabeled leukocyte scan with 99mTc-hexamethylpropyleneamineoxime has a high sensitivity and specificity in detecting diabetic foot infection [16], although MRI remains the preferred modality for comprehensive assessment of suspected extremity infection in most cases. FDG PET/CT is a promising imaging technique for detecting infection in the diabetic foot, but limited data exists for its efficacy.

MRI plays a crucial role in the examination of lower extremity infections, especially in cases of osteomyelitis, as it can provide precise details on both soft tissue and bone involvement. It is considered the most precise imaging technique for suspected pedal osteomyelitis, with a meta-analysis demonstrating a sensitivity of 90% and specificity of 79% [17]. However, certain factors such as prior surgery, neuropathic osteoarthropathy, or other inflammatory diseases such as rheumatoid arthritis could decrease the specificity of MRI [16]. The continued developments in imaging techniques, combined with a deeper understanding of these pathologies, hold promise in overcoming such potential diagnostic obstacles in the future. Consensus recommendations from the Society of Skeletal Radiology advise the use of contrast-enhanced MRI for the evaluation of infection in infants for improved detection of infection of unossified bone and in adults to highlight devitalized tissue. It is also recommended to evaluate for superimposed infection in the setting of neuropathic arthropathy, to assess complications of subacute and chronic osteomyelitis by highlighting ulcers and sinus tracts, and to detect bone and soft tissue abscesses [18].

The American College of Radiology (ACR) also offers ACR Appropriateness Criteria® for the imaging evaluation of Suspected Osteomyelitis, Septic Arthritis, or Soft Tissue Infection (Excluding Spine and Diabetic Foot) and Suspected Osteomyelitis of the Foot in Patients with Diabetes Mellitus that is helpful for classifying each imaging modality as “usually,” “may be,” or “usually not” appropriate for specific clinical indications and situations [19, 20].

## Cellulitis

Cellulitis is an acute nonnecrotizing infection of the skin, subcutaneous tissues, and superficial fascia. It is a common soft tissue infection most often affecting the lower extremities, usually below the knees [21, 22]. It presents clinically as soft tissue swelling, redness, warmth, and tenderness [21]. Regional lymphadenopathy is commonly present. Systemic signs and symptoms such as fever are not required for diagnosis but may indicate more severe infection [23, 24].

The most common route of entry of inoculating pathogens is through a skin break (open wounds and cutaneous lesions). *Streptococci* and *Staphylococcus aureus* (methicillin sensitive followed by methicillin resistant) are the most common organisms [23–25]. Other organisms like gram negative bacilli, anaerobes, and mycobacteria are often seen in immunocompromised patients [22]. Risk factors include diabetes, obesity, vascular insufficiency, chronic edema, immunocompromised states, and retained foreign bodies [23, 25]. Uncomplicated cellulitis is treated with oral or intravenous antibiotics; however, once abscess develops, incision and drainage or percutaneous drainage may be needed to establish source control [24].

As cellulitis is typically considered a clinical diagnosis, imaging is usually not necessary. Imaging is typically obtained in situations of rapid progression, severe systemic symptoms, or treatment failure. Imaging can also be utilized to search for foreign bodies or when complications are suspected [22, 23, 26, 27]. However, it is important to consider that many conditions which manifest with noninflammatory subcutaneous edema (bland edema), including generalized edematous states secondary to cardiac, liver, or renal failure, and unilateral lower extremity edema related venous or lymphatic stasis, may be difficult to distinguish from cellulitis radiographically. Unilateral asymmetric involvement in an extremity, clinical findings, and demonstration of hyperemia on color or power doppler and enhancement following intravenous contrast administration on CT or MRI can help distinguish cellulitis from bland edema [28]. Involvement of the deep fascia and muscles suggests an alternate or additional diagnosis, including nonnecrotizing and necrotizing fasciitis, abscess, or pyomyositis. Subcutaneous gas suggests necrotizing soft tissue infection [27]. There is no established role of diffusion weighted imaging in differentiating cellulitis from bland edema, but anecdotally, when intravenous administration of contrast on MRI is contraindicated or not possible, diffusion-weighted imaging may show small abscesses which may help differentiate the two [28]. Imaging can also detect foreign bodies, with CT being more sensitive than plain radiographs for radiopaque foreign bodies. US may also demonstrate foreign bodies as echogenic structures with posterior shadowing [27, 29].

Plain radiographs are often obtained to exclude soft tissue gas, which portends a more serious infection. More often, radiographs show nonspecific soft tissue swelling and increased density and/or reticulation in the subcutaneous tissues with loss of fat planes [27].

**Fig. 4:**
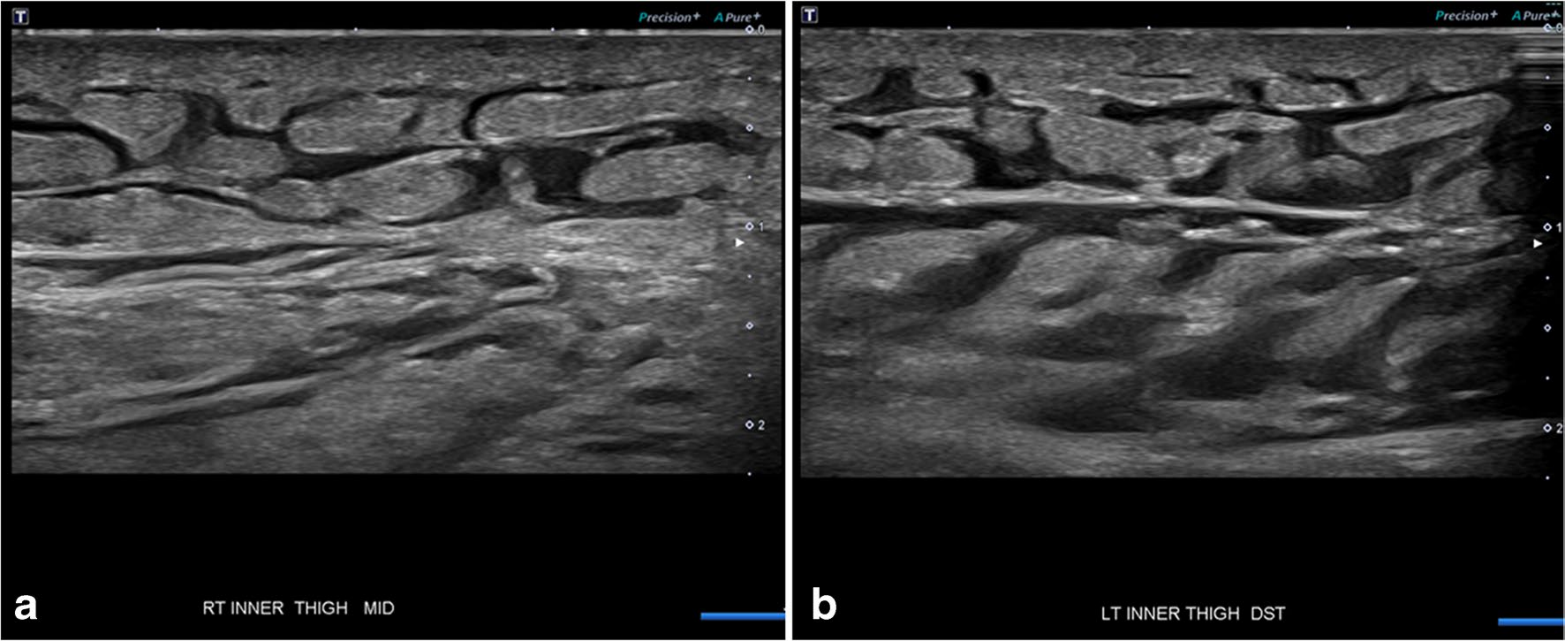
Sixty-seven-year-old man with renal failure and history of small bowel resection, total colectomy, and multiple intra-abdominal fluid collections presented with bland edema of the right (**a**) and left (**b**) upper inner thighs. Ultrasound shows “cobblestone” appearance. No focal fluid collection was seen

US is commonly obtained when an abscess is suspected or to exclude deep venous thrombosis [26]. In general, cellulitis and bland edema often demonstrate a “cobblestone” pattern related to echogenic subcutaneous fat lobules separated by thin insinuating fluid, with or without overlying skin thickening (Fig. 4). Increased vascularity is seen with color or power Doppler in cellulitis [22, 27, 29].

CT is often used in an emergent situation looking for soft tissue gas and other clues when necrotizing soft tissue infection is on the differential. Uncomplicated cellulitis may demonstrate skin thickening, edema, and/or stranding of the subcutaneous fat, and edema and thickening of the superficial fascia. As mentioned, diffuse enhancement or enhancing reticular septations in the subcutaneous fat is a feature that differentiates cellulitis from bland or noninflammatory edema. Abscess formation with or without the presence of gas can likewise be delineated [27, 30].

**Fig. 5:**
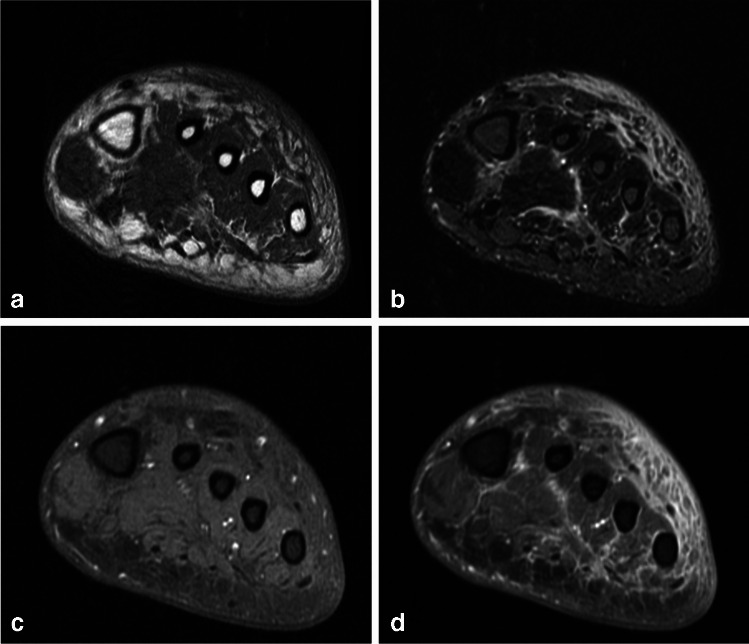
Thirty-five-year-old man with left foot pain, swelling, and erythema. Short axis T1 (**a**) and STIR (**b**) images show skin thickening with diffuse and reticulated low T1, hyperintense fluid sensitive signal limited to subcutaneous tissues consistent with subcutaneous edema. T1 FS images before (**c**) and following (**d**) administration of intravenous gadolinium-based contrast material shows diffuse and reticulated enhancement, correlating with his clinical diagnosis of cellulitis, and differentiating this condition from bland edema which would have no enhancement

MRI reflects similar changes to CT, including asymmetric subcutaneous edema and fat stranding with thickening of the overlying skin. Edema is seen as confluent or reticulated area of decreased signal intensity on T1 and hyperintense signal on fluid-sensitive sequences in the subcutaneous tissues and superficial fascia (Fig. 5) [15, 31–33]. As with CT, enhancement may be seen after intravenous administration of gadolinium-based contrast material [27], which distinguishes cellulitis from noninflammatory edema [25, 30]. Like CT, abscesses can be identified and delineated [25, 27]. On DWI, cellulitis appears as ill-defined diffuse and reticular hyperintensities with some restriction of diffusion on ADC map, while bland edema is seen as areas of increased diffusion [28].

## Abscess and phlegmon

An abscess is a walled-off collection of pus comprised of inflammatory cells, tissue fluid and debris, and microorganisms. Abscesses are likewise characteristically bounded by a peripheral capsule made up of inflammatory cells, fibrin, and granulation tissue [21, 22]. Phlegmon is its precursor, which is characterized as an infected inflammatory mass that can liquify and subsequently progress to abscess formation. It is ill-defined without a discrete wall and often surrounds an abscess [22, 34]. In the lower extremity, these are often a result of penetrating injury. Hematogenous spread can also occur, particularly in deep tissues. *Staphylococcus aureus* (most common), *Streptococcus pyogenes*, *Serratia marcescens*, and *Pseudomonas aeruginosa* are known causative organisms [15, 21, 22]. Less commonly, an abscess can be tubercular or fungal. Clinically, fluctuance will be present in addition to the other findings seen with cellulitis [31].

Imaging is often used for identification and localization of an abscess and to determine the path of drainage. It can be superficial, alongside, or deep to the investing fascia; within a muscle; or may span anatomical boundaries [33, 35]. Treatment options include open incision and drainage and image guided aspiration or drainage, with preference for the less invasive image guided approach in cases of diagnostic uncertainty [22, 36]. The Society of Skeletal Radiology recommends avoiding the use of the terminology “drainable” and “not drainable” in radiology reports [21]. Correlation with appropriate clinical setting is needed, as hematomas, seromas, ganglion cysts, and necrotic soft tissue tumors can mimic abscesses [37].

Plain radiographs usually show soft tissue swelling that may be mass-like. A gas-fluid level is uncommonly present and usually indicates pyogenic origin [15, 26].

**Fig. 6:**
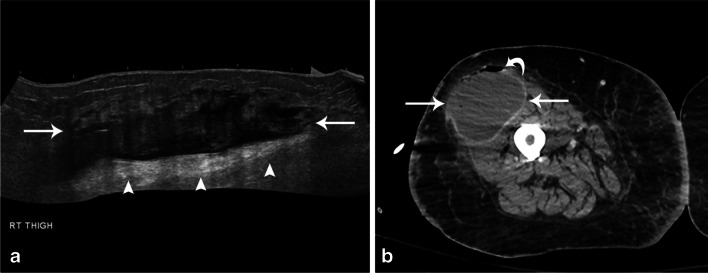
Fifity-six-year-old woman with right thigh pain and swelling. Extended view ultrasound image (**a**) of the right thigh shows an ill-defined heterogeneously hypoechoic collection (arrows) with posterior acoustic enhancement (arrowheads). The fluid collection was initially treated with antibiotics but did not resolve and subsequently was aspirated followed by placement of a drain. CT image (**b**) following placement of drain shows persistent hypodense collection with enhancing rim (arrows) consistent with an abscess. A gas-fluid level is seen (curved arrow). The specimen grew anaerobic gram-positive rods (*Clostridium* species, not *C. perfringens*) and *Enterococcus faecalis*

US is a useful tool to both evaluate a fluid collection and provide guidance for drainage. The appearance on US can be variable, ranging from an area that is centrally anechoic to hyperechoic, with pockets demarcated by a peripheral echogenic wall (Fig. 6a) [22, 37]. Posterior acoustic enhancement is usually seen and internal septations may be present [37]. Gas, seen as ill-defined echogenic areas with dirty shadowing, is uncommon but highly suggestive of an abscess [22, 37]. Mobile swirling debris seen with gentle pressure or change in position are also highly suggestive of abscess [33, 37]. On color or power Doppler, there should be no internal flow, though hyperemia may be present peripherally [22, 33]. US provides more detail of abscess contents than CT but the clinical importance of this has not been established [38]. Features such as posterior acoustic enhancement, motion of internal debris/purulent material, and lack of internal flow with or without hyperemia of the wall differentiates abscesses from solid or necrotic tumors [37].

On CT, an abscess appears as a centrally nonenhancing hypodense area bounded by thick, irregular, enhancing wall. Surrounding fat stranding/edema may be seen in superficial abscesses. In deeper or intramuscular abscesses, edema or enhancement of the surrounding musculature will be evident (Fig. 6b) [15]. Foci of gas or gas-fluid levels may also be present. CT is often used for guidance for aspiration and drainage of deeper abscesses. In one retrospective study that included abscesses in the lower extremity, US was found to be more sensitive, but CT more specific for diagnosis of superficial abscesses (sensitivity of 96.7% versus 76.7% and specificity of 85.7% versus 91.4% for US and CT, respectively) [21, 38].

**Fig. 7:**
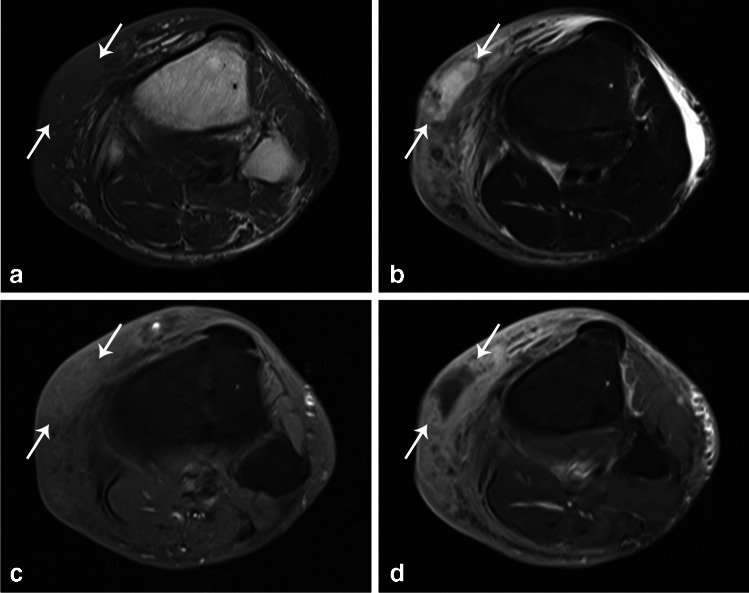
Forty-eight-year-old man with intravenous drug abuse presents with right lower leg pain, swelling, and redness. Axial T1 (**a**), STIR (**b**) and T1 FS pre- (**c**) and post-contrast (**d**) MRI images demonstrates an anteromedial subcutaneous fluid collection that is STIR hyperintense with rim enhancement (arrows) and adjacent enhancing cellulitis. Subsequent aspiration yielded cultures that were positive for *Staphylococcus aureus* and group A beta hemolytic *Streptococcus*

MRI is the imaging modality of choice for identification of soft tissue abscess and has a sensitivity and specificity of 89–97% and 77–80%, respectively [21, 33]. An abscess will appear as a focal, well-defined area of uniform to slightly heterogeneous high signal intensity on fat-suppressed fluid-sensitive sequences, with corresponding iso- to low signal intensity on T1-weighted sequences (Fig. 7) [35]. It is bounded by a rim of granulation tissue which is mildly hyperintense on T1 and slightly hypointense on fluid sensitive sequences compared to the central area, and will become more conspicuous following administration of intravenous gadolinium-based contrast material as the thick rim enhances, with central nonenhancement (Fig. 7) [35]. Gas, if present, is seen as signal void on all sequences. The “penumbra” sign, representing a rim of highly vascular, inflamed granulation tissue in subacute, chronic, or acute-on-chronic infections, is illustrated by relatively hyperintense signal on T1-weighted imaging surrounding the low signal abscess cavity, and has sensitivity and specificity of 54% and 98%, respectively. When present, it is useful for differentiating abscess from necrotic tumor [39]. On DWI, due to viscous internal pus, there is restricted motion resulting in high signal within the abscess cavity that will characteristically show low signal on corresponding ADC map images. Noncontrast-enhanced MRI with DWI has comparable sensitivity and specificity to contrast-enhanced MRI for diagnosis of soft tissue abscess [35]. Early abscesses with incomplete walls can be missed on contrast-enhanced MRI, while less viscous (i.e., liquified) abscesses may be missed on DWI imaging [35, 40]. In contrast, necrotic tumors classically will show restricted diffusion in the wall due to high cellularity as opposed to central restriction in abscess, though some necrotic tumors can mimic abscesses on DWI imaging [28]. With intramuscular abscesses, there will additionally be enlargement and edema of the musculature, but otherwise has a similar appearance to other soft tissue abscesses [33]. Intramuscular abscess is mimicked by diabetic myonecrosis, but again, DWI can help differentiate the two by showing restricted diffusion in the abscess cavity. Similarly, hematomas and seromas will not show restricted diffusion on DWI [28]. MRI is also useful for evaluation of osteomyelitis and septic arthritis that may occur concurrently [33].

Phlegmon has similar signal characteristics to an abscess on noncontrast MRI images and may or may not show the “penumbra” sign. On post contrast sequences, however, phlegmon will show diffuse or variable heterogeneous enhancement without a discrete enhancing rim. On ADC map images, phlegmon may show intermediate to high signal [21, 35]. On the other hand, bland edema in the subcutaneous tissues will show increased signal on both DWI and ADC map images, whereas devitalized tissue will show high signal on DWI and intermediate to low signal on ADC map images, respectively [35]. Within muscle, phlegmon is seen as edema-like fluid sensitive signal with architectural distortion or loss of muscle mass and increased motion on DWI [28]. There is, however, some overlap of findings on DWI between abscess and phlegmon, although ADC values tend to be lower with abscess [35].

## Necrotizing fasciitis

Necrotizing fasciitis is a rapidly progressive, limb and life-threatening infection. Necrotizing soft tissue infection is the preferred terminology, indicating infection associated with necrosis involving the skin and superficial soft tissues, fascia (superficial and deep), and muscles [21, 41]. It is associated with high morbidity (including amputation) and mortality [41, 42]. There is nearly four times the incidence of amputation in the lower extremities compared to the upper extremities [42]. A high index of suspicion and early surgical intervention is paramount for effective management [41].

Although necrotizing fasciitis affects all regions of the body, the lower extremities are the most common site of infection and demonstrate the fastest rate of spread [6, 25, 42]. Factors that increase the risk for necrotizing fasciitis include older age, male gender, diabetes, obesity, alcoholism and liver disease, malignancy, immunosuppression, and intravenous drug use [25, 42, 43]. The prevalence of diabetes in patients with necrotizing fasciitis is between 40 and 60% with a high incidence of necrotizing fasciitis coinciding with diabetic foot infection [43]. Other risk factors associated with necrotizing fasciitis, including vascular disease and renal failure, are also more common in patients with diabetes.

Most cases of necrotizing fasciitis are related to trauma which may be due to external injury (possibly innocuous) or recent surgery. In the lower extremities, it often originates from a skin infection, abscess, gangrene, or a surgical wound. Because of the proximity of the bones to the skin in the extremities, osteomyelitis is often associated with necrotizing fasciitis [43]. Synchronous multifocal necrotizing fasciitis involving more than one site occurs in 5% of cases and is also common in the lower extremities [44].

Necrotizing fasciitis can be divided according to the regions involved and the organisms responsible for the disease. Type 1 infections are polymicrobial, with both aerobic and anaerobic bacteria found on cultures. These predominantly affect patients with decreased immunity or chronic disease, and patients with uncontrolled diabetes are at particularly high risk. Type 2 infection is due to gram-positive monomicrobial associated with group A *Streptococcus* species, although *Staphylococcus* is also common. The virulence of ß-hemolytic streptococci activates the immune system resulting in a cytokine “storm” and toxic shock syndrome with multiorgan failure. Type 3 infection is gram-negative monomicrobial and is associated bacteria such as *Clostridium difficile*, or rarely *Vibrio*. *Clostridium* species infection is common with intravenous drug use [43]. Type 1 infections are most common, in one study accounting for more than half of cases [43]. Some authors have even included type 4 as a new category secondary to fungal infection (candida in immunocompromised and zygomycosis in immunocompetent patients) [43, 45].

Irrespective of the source of infection and causative organism, certain components of the pathophysiology are consistent. The infection starts in the hypodermis and the superficial fascia. There is thrombosis of the vessels and lymphatics in the subcutaneous tissues, which, along with compression of the vessels by edema, result in widespread necrosis. Rapid progression and systemic toxicity are the norm.

Erythema, local warmth, edema/swelling, and skin thickening are early manifestations, and systemic symptoms arise in later stages. There is often pain out of proportion to the area of involvement. Cutaneous bullae, often hemorrhagic in later stages, progress to necrosis. Crepitus is felt when subcutaneous gas is present from infection with gas forming organisms such as *Clostridium* species [45]. The clinical presentation may be nonspecific, especially in patients with diabetes and immunocompromise, which can result in a missed diagnosis. In patients with neuropathy related to diabetes or those who have taken or were given nonsteroidal anti-inflammatory medication, symptoms may be few or mild. These factors may consequently lead to a relatively higher rate of delayed diagnosis, increased mortality, and proximal amputation in these patient cohorts [42, 43].

The diagnosis is confirmed with surgical findings that show necrotic fat with brownish color fluid, lack of resistance to manual dissection, and tissue sampling showing necrosis. Bedside “finger test” (rapid finger sweep of the fascia through a small test incision) will show lack of bleeding in soft tissues, presence of brown “dishwater fluid,” and easy blunt dissection of the fascial planes [45–47].

Abnormal laboratory parameters are common. The Laboratory Risk Indicator for NECrotizing fasciitis (LRINEC) score, is a system compiling input aggregating C-reactive protein, white blood cell count, total hemoglobin, serum creatinine, sodium, and glucose level values, has been used to stratify risk (Table 1) [48]. A score of 6 or 7 out of a maximum score of 13 indicates moderate risk, with a score of 8 or greater representing high risk. For the lower extremity, the sensitivity and specificity for diagnosing necrotizing fasciitis are 49.3% and 83.17%, respectively [49, 50]. Combining the LRINEC score with MRI features such as thickening of the deep fascia (3 mm or more) and involvement of multiple compartments, shows improved positive and negative predictive values (82% and 79%, respectively) over LRINEC score alone (77% and 67%, respectively) in differentiating necrotizing from nonnecrotizing fasciitis [49].

**Table 1:** The laboratory risk indicator for necrotizing fasciitis (LRINEC) score

Laboratory test (units)	Value	Score
C-reactive protein (mg/L)	≤ 150	0
	> 150	4
White blood cell count (1000 cells/µL)	< 15	0
	15–25	1
	> 25	2
Hemoglobin (g/dL)	> 13.5	0
	11–13.5	1
	< 11	2
Sodium (mmol/L)	≥ 135	0
	< 135	2
Creatine (mg/dL)	≤ 1.6	0
	> 1.6	2
Glucose (mg/dL)	≤ 180	0
	> 180	1
Sum of scores = LRINEC score	< 6	Low risk
	6–7	Moderate risk
	> 7	High risk

The diagnosis of necrotizing fasciitis is predominantly clinical and aided by laboratory tests. Imaging is adjunct and valuable in equivocal cases, and in those who are at moderate risk. Despite the ionizing radiation and inferior soft tissue resolution compared to MRI, CT is often the preferred modality due to considerably shorter imaging time, its ability to detect soft tissue gas, and wider availability [51]. MRI provides the most information but can be lengthy and should not preclude early surgical intervention. The emphasis of imaging is twofold: to detect gas within the soft tissues and to determine involvement of the deep fascia [29]. CT or MRI can be used depending on the clinical situation and need for rapid diagnosis, with ultrasound reserved for use at institutions that have expertise in diagnosing necrotizing fasciitis using this modality.

**Fig. 8:**
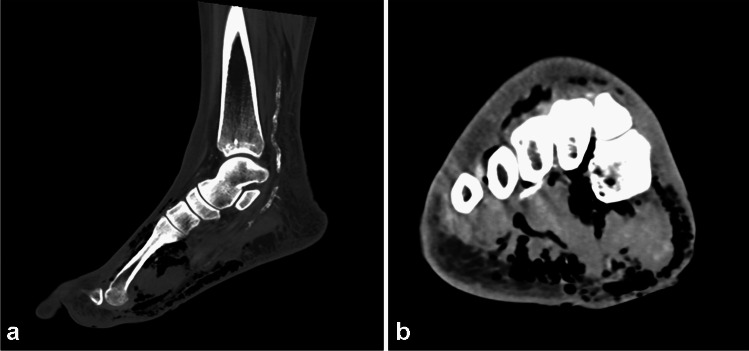
Sixty-four-year-old man who presented with right foot infection that had worsened acutely over the last several days. On clinical examination, there was necrotic great toe (black toe) with ascending erythema and desquamation, and crepitus. Apparent “woody” edema was noted in the proximal foot and distal ankle, with edema extending up to the knee. He had LRINEC score of 12. CT scan without contrast shows gas dissecting within the subcutaneous tissues and along the deep peripheral and deep intermuscular fascia. He was taken to the operating room where findings of necrotizing soft tissue infection were found, later confirmed pathologically. He had emergent amputation. Blood culture grew gram negative coccobacilli, *Prevotella* species, *Clostridium* species (not *C. perfringens*), *Parvimonas micra*, *Streptococcus agalactiae* (group B), and methicillin-resistant *Staphylococcus aureus* (MRSA)

Plain radiographs, CT, US, and MRI all can show gas in the soft tissues, with gas seen as hyperechoic foci with “dirty” posterior shadowing on US and foci of low signal intensity on all pulse sequences on MRI. Plain radiographs and CT are best to show the presence of gas within the soft tissues, but the sensitivity is poor. On plain radiographs, gas is seen only in about one fourth to one half of patients [6]. CT is more sensitive than plain radiographs for detection of gas, but it showed poor sensitivity in a recent systematic review (sensitivity and specificity of 48.6% and 93.2%, respectively) [52] (Fig. 8). Although very specific, failure to detect gas does not exclude the diagnosis, as gas is seen late in the disease [52, 53], and a lack of soft tissue emphysema does not exclude the diagnosis of necrotizing fasciitis [6, 54]. Foreign bodies can also be identified with imaging.

CT can show edema/fluid and thickening along the superficial and deep intermuscular fascia, fluid collections, and muscle necrosis [55]. In a retrospective study, a CT-based scoring system based on presence of perifascial air, muscle/fascial edema, fluid tracking in subcutaneous tissues, lymphadenopathy, and subcutaneous edema (points from 5 to 1, respectively) was developed by McGillicuddy et al. for distinguishing necrotizing soft tissue infections from nonnecrotizing soft tissue infections. Using a cutoff of more than 6 from a maximum score of 15, the scoring system showed sensitivity and specificity of 86.3% and 91.5% respectively for diagnosis of necrotizing soft tissue infection [51]. Another recent retrospective study with a small sample size of positive cases utilized the presence of at least one of the criteria of gas in the soft tissues, multiple fluid collections, absence or heterogeneity of tissue enhancement following intravenous contrast, or significant inflammatory changes under the fascia, and showed sensitivity and specificity of 100% and 97.7%, respectively [47]. However, further prospective studies are necessary to confirm the results. Other findings commonly seen in necrotizing infection include soft tissue swelling and osteomyelitis in the foot.

**Fig. 9:**
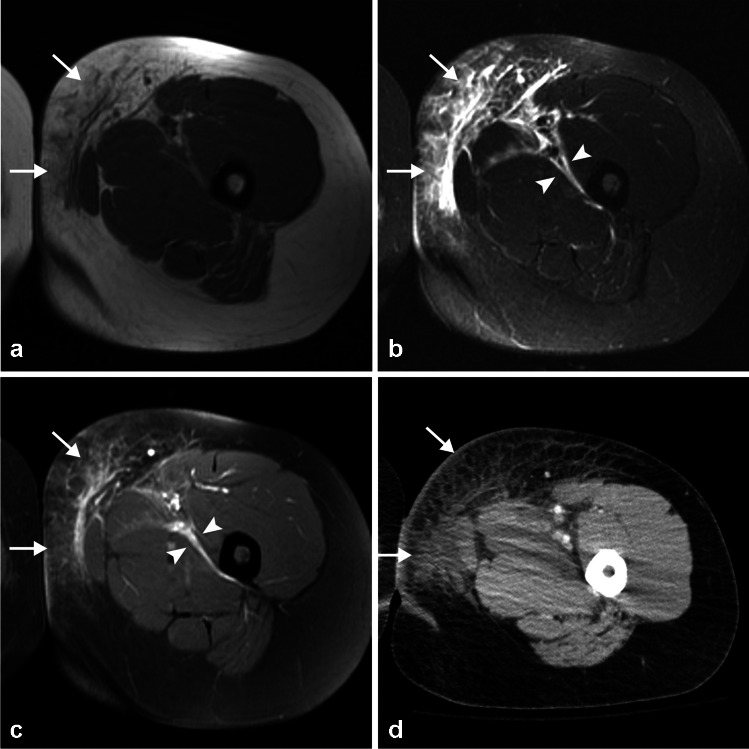
Twenty-two-year-old woman with left groin and thigh pain for 6 days. Axial T1 (**a**) and STIR (**b**) images show thickening and fluid along the deep peripheral fascia overlying the adductor and extensor compartments of the thigh (arrows) with extension along the deep intermuscular fascia between the two compartments and the muscles of the adductor compartment (arrowheads). T1 FS image following the administration of intravenous gadolinium-based contrast (**c**) shows enhancement of these fasciae. There is also edema and enhancement within the overlying subcutaneous tissues with thickening of the skin. Two days later, there was no improvement in patient’s condition and a CT scan with contrast (**d**) was performed which show similar findings. Necrotizing fasciitis was confirmed at surgery

On MRI, edema along the deep fascia is the most commonly used criteria (Fig. 9). Involvement of deep fascia in 3 or more compartments, greater than or equal to 3 mm thickening, and extensive involvement were more common in necrotizing soft tissue infection compared to nonnecrotizing soft tissue infection and showed high sensitivity and specificity. Fascial enhancement is variable such that the fascia may or may not enhance, or may show a mixed pattern [25]. Lack of enhancement of the fascia on postcontrast fat-suppressed T1-weighted sequences can be seen with both necrotizing fasciitis and nonnecrotizing soft tissue infections, representing either necrotic fascia in necrotizing fasciitis or nonenhancing perifascial fluid collection in nonnecrotizing soft tissue infection [54]. However, MRI can both overestimate the disease by showing reactive edema and enhancement in adjacent noninfected fascia or underestimate the disease by showing lack of enhancement secondary to occlusion of vessels or necrosis [29, 31, 32]. Nevertheless, MRI can help exclude the disease with high specificity when deep abnormality is absent [21, 32]. DWI does not help in the diagnosis but can highlight the presence of concomitant abscesses [28].

Ultrasound has been used in diagnosis of necrotizing fasciitis and may show nonspecific thickening and fluid along the fascia [6, 56, 57]. Although promising initially, a more recent retrospective study could not reproduce the high sensitivity using a similar cutoff and instead proposed a cutoff of 2 mm, with sensitivity and specificity of 75% and 70.2%, respectively [57]. Although ultrasound can show findings suggestive of necrotizing fasciitis, CT and MRI are far superior to ultrasound for evaluation of deeper structures.

Differential considerations for necrotizing fasciitis include noninfectious fasciitis (including paraneoplastic fasciitis, eosinophilic fasciitis, and nodular and proliferative fasciitis), pyomyositis, diabetic myonecrosis, cellulitis with thrombosis, and compartment syndrome. Other noninfective conditions such as dermatomyositis, lupus myofasciitis, Churg-Strauss vasculitis, and graft versus host disease, may likewise have similar overlapping imaging features [6, 21, 53].

## Pyomyositis

Pyomyositis represents an uncommon infectious condition that causes suppuration within striated muscles. Historically, this condition has predominantly been diagnosed in the tropics. However, in more recent decades, a steady increase incidence has been observed even outside of those regions, particularly in Europe and the USA [58]. Although this condition can occur at any age, its incidence is more frequent in the pediatric population [59].

An early diagnosis is essential for saving tissue and the patient’s life, but it is frequently overlooked due to the absence of early specific indicators, unfamiliarity with the disease among physicians, atypical clinical presentations, and a large range of possible differential diagnoses. Due to this, imaging studies play a key role in the recognition of the condition. Moreover, radiological studies are fundamental in follow-up, image-guided interventional procedures (drainage/aspiration), and treatment response evaluation [29].

**Fig. 10:**
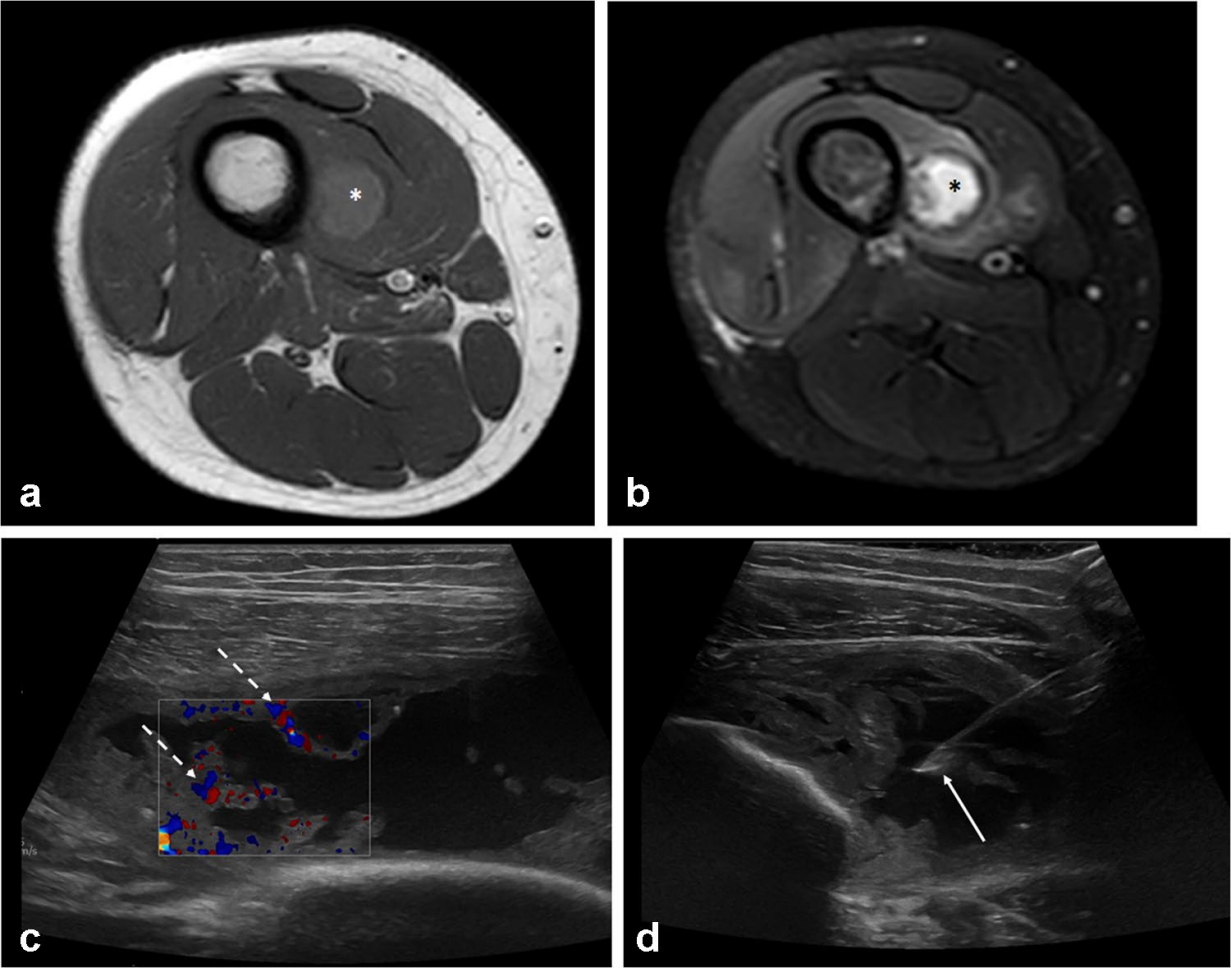
Twelve-year-old boy with right thigh pain and swelling. T1 (**a**) and T2 FS (**b**) MRI images revealed features of pyomyositis in the abscess phase, within the vastus intermedius muscle (asterisks). A mild hyperintense signal intensity on T1 sequence can be noted, possibly related to the proteinaceous content of the infected fluid-collection. Marked hyperemia of the abscess walls can be detected on color-Doppler ultrasound (**c**, dotted arrows). The patient underwent ultrasound-guided aspiration (**d**) with an 18G needle (arrow), for microbiological assessment. The diagnosis of methicillin-resistant *Staphylococcus aureus* infection was obtained

Pyomyositis is subdivided into primary pyomyositis produced by the hematogenous transmission of several possible microorganisms, and secondary pyomyositis that occurs when the muscle is infected through contiguous tissues, such as a bone, joint, or soft tissue. *Staphylococcus aureus* is the most prevalent type of bacteria to cause infectious pyomyositis; tuberculous and nonbacterial pyomyositis caused by viruses, fungi, and parasites are uncommon. Methicillin-resistant *Staphylococcus aureus* is an emerging infectious disease that deserves special consideration and may affect both adults and children (Fig. 10). The capacity of this pathogen to create toxins, such as Panton-Valentine leukocidin, which has a special ability to kill leukocytes and allows bacteria to evade the bactericidal action of leukocytes, may contribute to its virulence. In the early stages, symptoms including limping, hip discomfort, and fever might be modest, which can delay diagnosis. Most frequently, the thighs, calves, and pelvic muscles are affected.

Conventional radiography may show focal soft tissue swelling, exclude or confirm bone involvement, or detect radiopaque foreign bodies. Ultrasonography can be used as the initial diagnostic step to rule out other potential causes of limping, such as septic arthritis. However, ultrasound imaging has a limited sensitivity when it comes to early infection and deep abscess localization. Contrast-enhanced CT can demonstrate enlarged muscles, uneven attenuation, gas components, and a central fluid collection with rim contrast enhancement, but it is ineffective in identifying the early stages of pyomyositis and detecting bone involvement [29, 59]. US and CT are both useful for guidance for aspiration or drainage for diagnosis of the infecting microorganism and determination of antibiotic susceptibilities. Contrast-enhanced MRI is considered the most sensitive and accurate imaging tool for the assessment of the disease. Indeed, MRI can accurately evaluate deep and superficial soft tissues as well as bone.

MRI offers important help in narrowing the differential diagnosis (e.g., soft-tissue sarcomas, soft-tissue lymphomas, cellulitis) and may help to identify the pathogen involved. Contrast media injection is suggested for diagnosis and diffusion-weighted imaging (DWI) may be very helpful. DWI can show water motion restriction inside the collection due to viscous pus with a low apparent diffusion coefficient (ADC = 0.6–0.11 × 10^−3^ mm^2^/s), which can be useful to distinguish these infections from a highly necrotic soft tissue neoplasm. Higher fluid-like ADC values (> 2 × 10^−3^ mm^2^/s) are displayed by more liquefied intramuscular infections in later stages [60]. In the early phlegmonous stage, MRI will demonstrate muscle enlargement, T2 hyperintensity, and loss of the normal muscle fiber architecture without a well-defined rim. In the late stage, MRI will demonstrate a T1 iso- or hypointense, T2 hyperintense, rim enhancing intramuscular abscess.

Minimally invasive fluid sampling for microbiological diagnosis can be extremely helpful in clinical practice, though patients may benefit from treatment prior to microbiological analysis or even with negative microbiological tests. One of the easiest and most accurate evaluations feasible on MRI is the distinction between viral and bacterial myositis. Streaky or patchy infiltration of the muscle and excessively high signal on T2-weighted sequences are typical findings of the more uncommon viral types [61]. One series by Thammaroj et. al found that hyperintense signal on fluid-sensitive MRI sequences within the abscess wall was significantly correlated with bacterial pyomyositis as opposed to tubercular, although further validation is needed [62]. In the future, MRI-based radiomics analysis promise a more accurate microbiological diagnosis contributing to more tailored care.

## Infectious tenosynovitis

Infectious tenosynovitis, and similarly infectious bursitis, can become an orthopedic emergency if not properly recognized and treated. The tendon sheath and bursal wall act as a sealed compartment which is susceptible to infection. Inflammation, trauma, and infection can all lead to tenosynovitis. Infectious tenosynovitis can cause rigidity of the affected tendon tissues and lead to permanent impairment. It is therefore vital to identify this entity in a timely fashion and distinguish it from its noninfectious counterpart, though this can be a diagnostic challenge. Skin trauma that introduces a pathogen is usually the starting point of these conditions [29].

*Staphylococcus aureus* is the microbe most frequently associated with infectious tenosynovitis, followed by other bacteria including *Pasteurella multocida* (typically in cat bites), *Neisseria gonorrhoeae* (sexually transmitted), and *Eikenella corrodens* (typically in human bites). Infectious tenosynovitis caused by *Mycobacterium tuberculosis* can also occur and typically presents subtly with progressive swelling and variable exam findings. *Mycobacteria marinum* from exposure to open wounds in the sea is another mycobacteria that can be responsible for this condition [63].

In the event of a delayed diagnosis, conventional radiography can be utilized to rule out the existence of retained foreign bodies or to demonstrate osseous involvement (erosions and/or periosteal reaction). US can help to identify aberrant synovial hyperplasia and increased fluid and debris in the tendon sheaths. Hypervascularity may be present or absent by power Doppler or color Doppler evaluation. US also allows for immediate diagnostic evaluation with US-guided biopsy or aspiration.

**Fig. 11:**
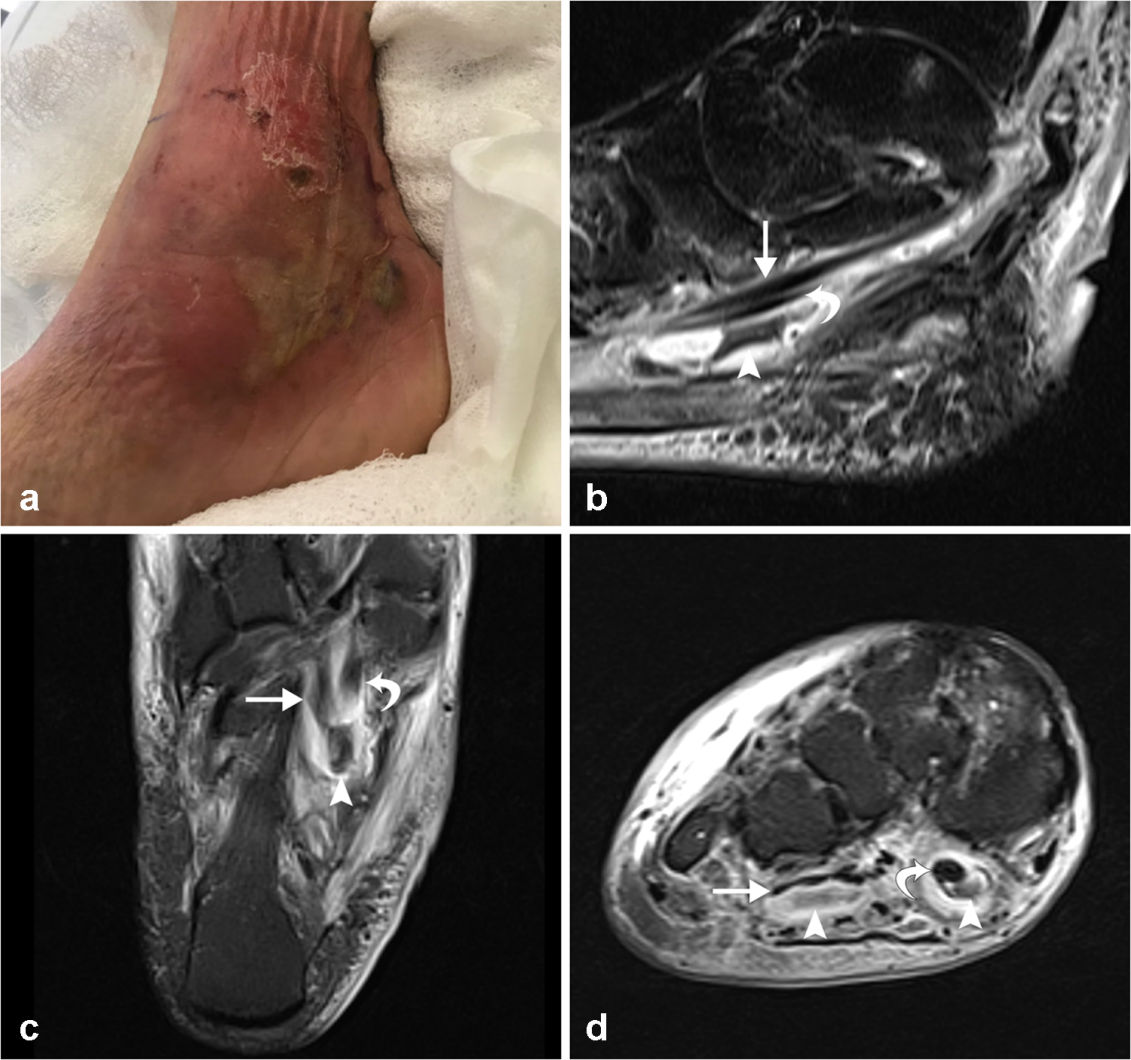
Seventy-five-year-old man with right medial ankle redness, swelling, and ulceration. Clinical photograph (**a**) demonstrates medial ankle cellulitis and skin breakdown. Sagittal (**b**), long axis (**c**), and short axis (**d**) STIR MR images demonstrate moderate to severe infectious tenosynovitis of the flexor digitorum longus (arrows) and flexor hallucis longus (curved arrows) tendons and at the Master knot of Henry with intermediate to hypointense debris (arrowheads) within the tendon sheaths

Tendon thickening, fluid accumulation, or abscess might be seen on a CT scan, particularly after the intravenous injection of contrast media. Above all, MRI is the most accurate and focused imaging technique. The increased fluid build-up within the tendon sheaths can be readily detected by MRI, which can also provide a useful assessment of synovial hyperplasia (Fig. 11). Paratendinous contrast enhancement can also be seen on a gadolinium-enhanced MRI as a result of widespread inflammation. “Rice bodies” represent small (2–7 mm) rounded foci of acellular fibrinous material that are typically T1 hypointense and slightly T2 hyperintense within a synovial lined structure. Although this finding is nonspecific, it may be seen with infectious tenosynovitis, especially with tuberculous or fungal infection. Differential diagnosis includes inflammatory tenosynovitis (e.g., with rheumatoid arthritis), idiopathic tenosynovitis with rice bodies, pigmented villonodular synovitis, and synovial chondromatosis. The presence of gas, an ulcer or skin breach, foreign body, overlying cellulitis or soft tissue edema, and concomitant septic arthritis favor infectious tenosynovitis over the alternative diagnoses [31]. Imaging alone is frequently insufficient to make the diagnosis, and US-guided aspiration and/or biopsy with microbiological analysis is necessary to establish a definitive diagnosis.

## Septic arthritis

**Fig. 12:**
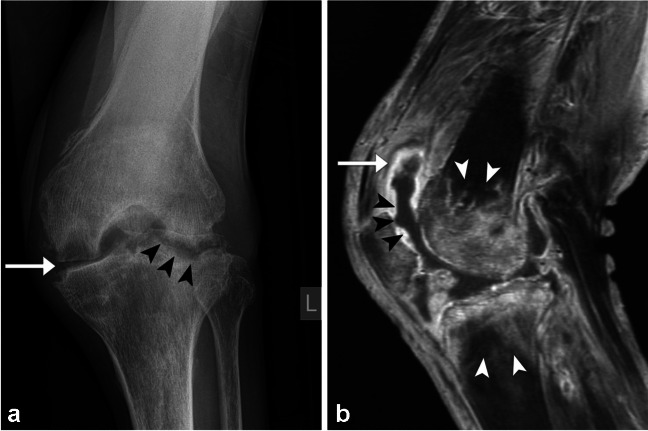
Frontal projection radiograph of the left knee (**a**) in a 60-year-old male with known septic arthritis demonstrating severe joint space narrowing (white arrow), in addition to periarticular osteopenia and marginal erosions with central erosions (black arrowheads). Sagittal T1-weighted post-contrast MR image of the left knee with fat-suppression (**b**) in a 79-year-old with an established diagnosis of septic arthritis showing avidly enhancing, thickened synovium (white arrow) with complex, heterogenous joint effusion (black arrowheads). Additional findings of confluent, abnormal marrow enhancement in the distal femur and proximal tibia (white arrowheads) are compatible with concomitant osteomyelitis

In the absence of surgical or traumatic history, septic arthritis classically presents as a monoarticular arthropathy caused by hematogenous seeding of the synovial fluid by pathogenic bacteria, with a predilection for large joints such as the hip and knee [14, 64–68]. Marked inflammatory changes at the articular surface lead to clinical features of joint pain, swelling, and erythema, and may rapidly progress to irreversible cartilage destruction and even death [14, 66–69]. Radiologic imaging plays a crucial role in differentiating septic arthritis from more benign or chronic processes and is of utmost diagnostic utility in cases where synovial fluid cannot be adequately sampled for culture [7, 14, 66, 67]. Conventional radiography, while notably insensitive for early changes, is often the first imaging obtained. Images may show nonspecific findings of soft tissue swelling and joint effusion, the latter of which may lead to joint space pseudo-widening if sufficiently large [64–66, 68]. Marginal erosive changes with loss of the normal subchondral bone plate, periarticular osteopenia, and narrowing of the joint interline signal more advanced disease (Fig. 12a) [7, 68, 69]. If left untreated, findings may progress and lead to permanent joint malalignment and ankylosis [65, 66, 69].

Ultrasound serves a complementary diagnostic role, whereby the absence of joint effusion has a high negative predictive value to rule out joint space infection [66]. Cross-sectional imaging may show similar features as described above, though has greatest utility in delineating extent of disease and assessing for complications [7, 65, 67, 68]. MRI in particular is highly sensitive and may reveal findings of synovial thickening and enhancement, cartilage destruction, and subchondral/perisynovial edema, in addition to complex joint effusion with internal debris [7, 64–66, 69]. Edema signal and post-contrast enhancement extending to the medullary bone should prompt consideration of concomitant osteomyelitis, while the presence of complex fluid and enhancement within tendon sheaths raises concern for concomitant tenosynovitis (Fig. 12b) [7, 64]. In cases of diagnostic uncertainty, nuclear medicine exams such as bone and leukocyte labeled scintigraphy may help to localize infectious foci and simultaneously assess for the presence of multifocal disease, though these studies are limited by poor specificity, prolonged acquisition times, and high cost [67, 68, 70].

## Osteomyelitis

Osteomyelitis (OM) is defined as an infection of the bone involving the medullary space [21]. The most common cause is direct inoculation from an adjacent skin defect [12, 34, 71, 72]. In cases of the lower extremity, this is usually a complication of diabetes with superimposed peripheral neuropathy and vasculopathy [72]. Another mode for direct inoculation is penetrating trauma. Patients with open fractures are susceptible to polymicrobial OM, similar to patients with diabetic foot ulcerations [71]. As opposed to direct inoculation, hematogenous OM is a result of bone seeding and is a complication of bacteremia or atypical lung infection in patients who are immunocompromised [71]. Hematogenous OM is more common in children and has a propensity for the well-vascularized axial skeleton [12, 34]. When affecting the peripheral skeleton in adult patients, OM tends to occur in the metaphysis due to slow moving blood flow in the terminal capillaries [12].

Osteomyelitis can also be defined in terms of chronicity. Symptoms that have been present for under two weeks are typically classified as acute whereas chronic osteomyelitis lasts 4 weeks or longer [21]. Acute and chronic osteomyelitis differ with regards to clinical presentation, imaging findings, and treatment.

Patients with acute OM resulting from direct inoculation often present with systemic symptoms, such as fever and chills, as well as localized pain, swelling, and erythema [72]. Purulent drainage from an open wound and the ability to probe the underlying bone are highly suggestive, but not diagnostic of OM [21, 73]. The clinical presentation of chronic and hematogenous OM is more widely variable and symptoms may be absent [72, 73].

Patients suspected to have OM should have laboratory tests assessing white blood cell (WBC) count, erythrocyte sedimentation rate (ESR), and C-reactive protein (CRP) [73]. Blood cultures may be considered, but tend to be most contributory in hematogenous OM [12, 73]. In one study of 90 patients with OM and diabetic foot ulcer, an ESR value of 53.5 mm/h yielded a sensitivity and specificity of 84% and 91%, respectively. A CRP level of 5.19 demonstrated a sensitivity of 87% and specificity of 73% [74]. Another study of 353 diabetic patients with infection determined cut off values of 60 mm/h and 7.9 mg/dL for ESR and CRP, respectively, above which the likelihood of osteomyelitis was high [75]. Additionally, the authors concluded that there was low likelihood of OM with an ESR value of less than 30 mm/h [75].

The gold standard for diagnosing OM is a positive culture from the site of infection [73, 76]. However, reported rates for culture positivity are low, ranging from 21 to 60%, and therefore, biopsy for the sole purpose of organism isolation should be performed with restraint [21, 76]. If biopsy is performed, it is recommended to discontinue antibiotic therapy prior to the procedure [73].

Many imaging modalities have the potential to diagnose OM. In any setting of suspected lower extremity infection, conventional radiography is recommended as the initial imaging test of choice despite its relatively low sensitivity [73, 77]. The earliest sign of OM on radiography is osseous demineralization secondary to hyperemia [71].

**Fig. 13:**
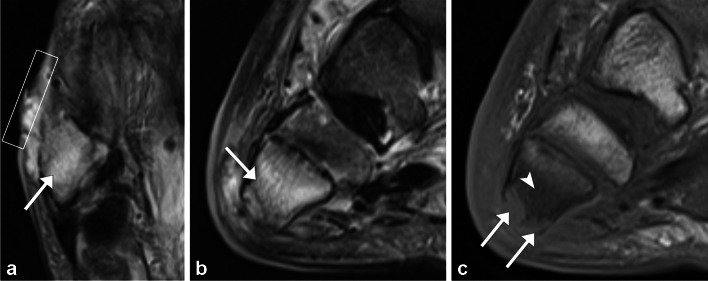
Seventy-one-year-old diabetic man with fifth ray infection status posttransmetatarsal amputation with positive margins. Long (**a**) and short (**b**) axis STIR images through the foot obtained several weeks after surgery demonstrate bone marrow edema like signal intensity approaching that of fluid within the fifth metatarsal stump (arrows). There is adjacent skin irregularity and abnormal T2 hyperintense subcutaneous signal (rectangle in **a**) without definite sinus tract. Short axis T1 MR image (**c**) shows cortical irregularity (arrows) and confluent medullary hypointense signal (arrowhead) within the fifth metatarsal stump. This patient underwent complete fifth metatarsal excision with pathology showing bony remodeling and chronic osteomyelitis with adjacent chronically inflamed soft tissue

**Fig. 14:**
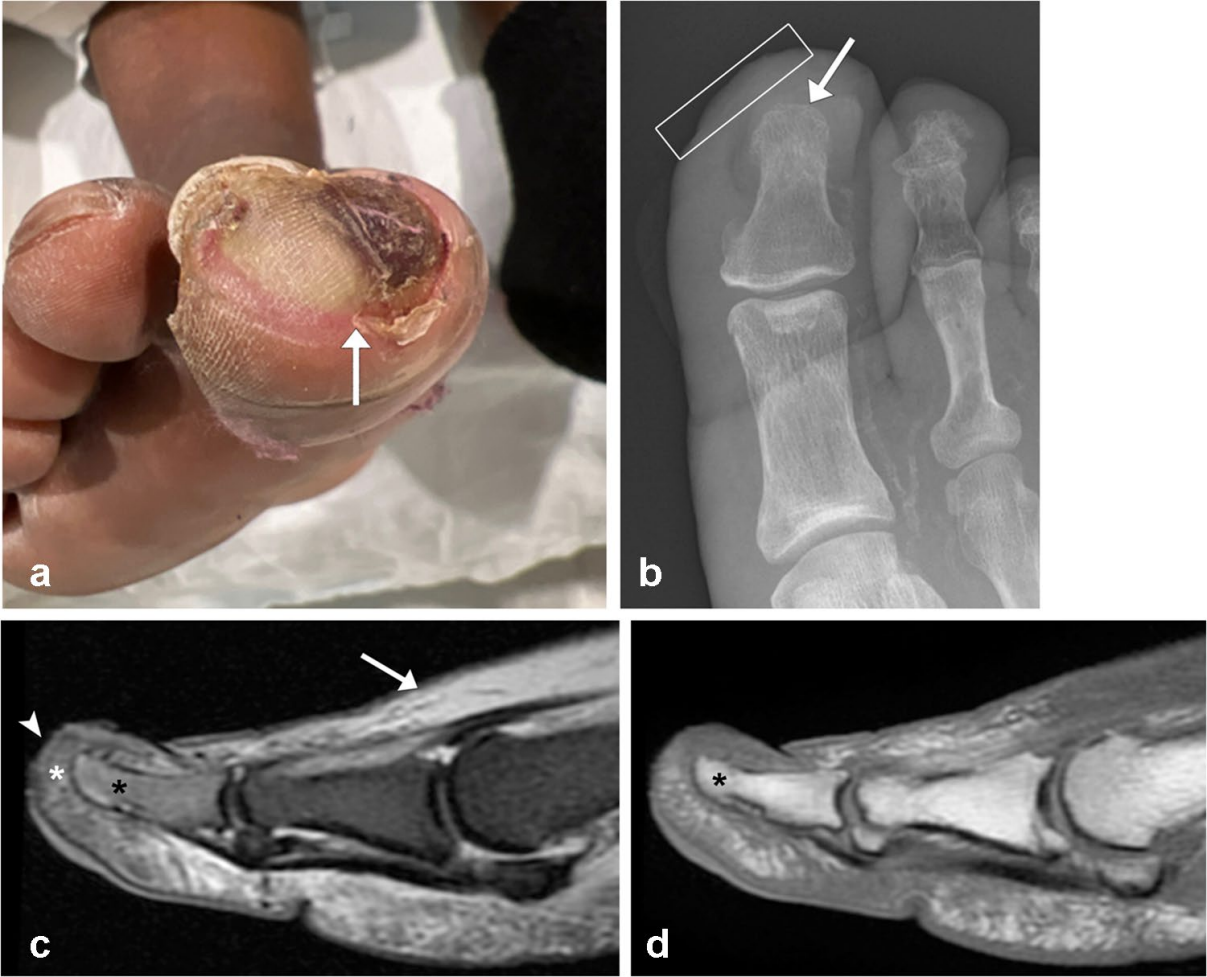
Fifty-four-year-old diabetic woman with right great toe infection. Clinical photo (**a**) illustrates an ulceration of the distal great toe (arrow). There is no exposed bone. Frontal radiograph (**b**) of the right great toe shows subtle skin irregularity corresponding to patient’s known ulcer (rectangle). The underlying bone demonstrates normal bone mineral density without cortical disruption (arrow). Sagittal STIR MR image (**c**) shows T2 hyperintense signal throughout the first distal phalanx (black asterisk) which is significantly lower in signal compared to the adjacent subcutaneous edema (arrow). There is a preserved fat plane (white asterisk) between the ulcer (arrowhead) and the bone. Sagittal T1 MR image (**d**) shows preservation of the marrow fat (asterisk). This patient was diagnosed with cellulitis and reactive bone marrow edema and discharged with a course of oral antibiotics

In the acute setting, imaging findings on MRI consist of bone marrow edema, with signal approaching that of fluid and geographic, confluent marrow replacement on T1-weighted imaging, with signal isointense or hypointense relative to skeletal muscle (Fig. 13) [34, 66, 78]. The presence of cortical irregularity, cortical erosion, and periosteal reaction are also suggestive of the diagnosis [12, 66, 71, 78]. In general, the probability of OM greatly increases when soft tissue infection (ulcer and/or sinus tract) directly abuts the abnormal bone [21, 66]. Figure 14 illustrates an example of signal alteration which is not diagnostic of osteomyelitis. In acute OM, the affected bone will typically enhance with the administration of intravenous contrast, but it is not required nor recommended to diagnose acute OM [73]. Contrast may be helpful in visualizing adjacent soft tissue infection, sinus tracts, and abscesses [71, 77]. The treatment for uncomplicated acute OM is antibiotics [76].

Recent advancements in CT imaging, specifically dual energy CT (DECT), have made it possible to detect bone marrow edema on CT using spatially matching data sets acquired simultaneously or sequentially at different tube voltages, usually 70–80 kVp and 140–150 kVp [79]. The data can then be post processed using a virtual noncalcium algorithm, differentiating healthy bone marrow which has a higher fat content from edematous marrow which has a higher water content and Hounsfield unit measurement [80]. Preliminary studies have shown promising results in the ability of DECT to detect lower extremity osteomyelitis, with one study showing comparable diagnostic efficacy to MRI, particularly when the virtual noncalcium images are read in conjunction with standard CT bone and soft tissue reconstructions [80–82]. Advantages of DECT include lower cost, faster acquisitions times, and virtually no contraindications [80]. Disadvantages include the difficulty in picking up subtle findings on DECT in the smaller bones of the midfoot and forefoot [80]. In terms of soft tissue evaluation, CT allows for easier detection of soft tissue gas when compared to MRI. Though the soft tissue contrast resolution in DECT is superior to multi-detector CT, MRI remains the preferred modality for evaluation of soft tissue involvement in infection [79, 81].

**Fig. 15:**
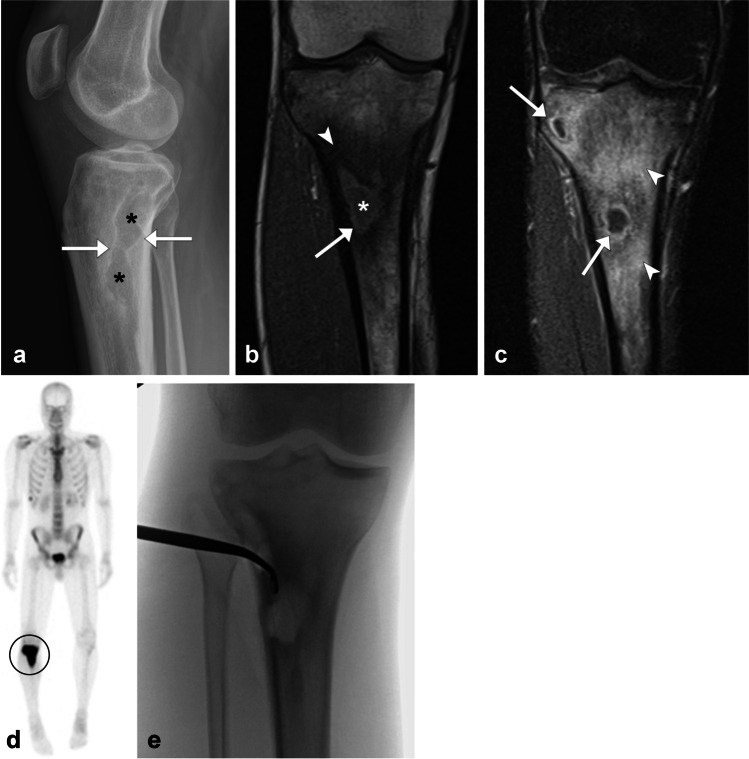
Twenty-seven-year-old man with chronic hematogenous OM. Lateral radiograph of the knee (**a**) demonstrates an irregular area of lucent bone (asterisks) with surrounding reactive sclerosis (arrows). Coronal T1-weighted MR image (**b**) of the proximal tibia shows an intraosseous abscess (asterisk) with hyperintense rim or penumbra sign (arrow) and surrounding reactive sclerosis (arrowhead). Coronal T1 postcontrast fat saturated MR image (**c**) highlights additional smaller abscesses with peripheral enhancement (arrows) and extensive surrounding bone marrow enhancement (arrowheads). Whole body bone scan (**d**) demonstrates intense uptake in the proximal right tibia (circle). Fluoroscopy image (**e**) was performed intraoperatively at which time approximately 20 mL of purulent material was aspirated from three pockets. Frozen section showed a mixed inflammatory infiltrate

The hallmark of chronic OM is the formation of a sequestrum, defined in this context as a piece of necrotic and sclerotic bone surrounded by granulation tissue, which serves as a nidus for infection [71, 83]. In response, the body forms reactive new bone (involucrum) and an opening in the cortex through which to push out the infection (cloaca) [21, 71]. A cloaca may contribute to the formation of subperiosteal abscess [12]. Intramedullary, or Brodie’s, abscess may also be found in subacute to chronic OM (Fig. 15) [12, 66, 69]. The penumbra sign has been described for an abscess consisting of a thin hyperintense rim on noncontrast T1-weighted sequences [21]. CT may be useful in the setting of chronic OM to identify sequestra [21, 71, 73]. On MRI, the medullary cavity can show areas of patchy involvement with superimposed fibrotic changes and cortical remodeling [21]. Antibiotics may not have access to the sequestrum; therefore, the treatment of chronic OM typically involves surgery [12, 66, 72].

Imaging findings in hematogenous OM can be nonspecific with aggressive appearing bony destruction and periosteal reaction on radiography (Fig. 15) [69]. Due to the loose adherence of the periosteum to the underlying bone in children, subperiosteal spread of infection may occur [21, 69]. Pathologic fractures are a known complication [69]. The treatment for uncomplicated hematogenous OM is intravenous antibiotics [84].

## Conclusion

Lower extremity infection in an increasingly common cause of morbidity among patient presenting for medical care and as a hospital acquired infection. It is critical for the radiologist to have an understanding of the characteristic imaging features of cellulitis, abscess versus phlegmon, necrotizing soft tissue infection, pyomyositis, infectious tenosynovitis, septic arthritis, and osteomyelitis in order to be able to differentiate these conditions from noninfectious causes of swelling and edema.
